# Predictors of survival among Japanese patients receiving first‐line chemoimmunotherapy for advanced non‐small cell lung cancer

**DOI:** 10.1111/1759-7714.13720

**Published:** 2020-10-30

**Authors:** Yuri Ogura, Nobutaka Kataoka, Yusuke Kunimatsu, Yusuke Tachibana, Takumi Sugimoto, Nozomi Tani, Izumi Sato, Kazuki Hirose, Daishiro Kato, Takayuki Takeda

**Affiliations:** ^1^ Department of Respiratory Medicine Japanese Red Cross Kyoto Daini Hospital Kyoto Japan; ^2^ Department of Thoracic Surgery Japanese Red Cross Kyoto Daini Hospital Kyoto Japan

**Keywords:** Advanced lung cancer inflammation index, chemoimmunotherapy, neutrophil‐to‐lymphocyte ratio, non‐small cell lung cancer, prognostic nutrition index

## Abstract

**Background:**

First‐line chemoimmunotherapy (CIT) has improved overall survival (OS) and progression‐free survival (PFS) outcomes among patients with non‐small cell lung cancer (NSCLC). The immunological and nutritional statuses of patients fluctuate during treatment using immune checkpoint inhibitors, and are closely related to treatment outcomes. However, it is unclear whether these markers are significant in patients who are receiving CIT.

**Methods:**

This retrospective single‐center study evaluated 34 consecutive Japanese patients with NSCLC who were treated using first‐line CIT. Previously reported markers that reflect immunological and nutritional statuses were evaluated at three time points: at the start of CIT, after three weeks, and at the end of induction therapy.

**Results:**

The median PFS was 7.2 months (95% confidence interval: 6.3 months–not reached) and the median OS was not reached (95% confidence interval: 9.6 months–not reached). The PFS duration was significantly associated with the baseline neutrophil‐to‐lymphocyte ratio and the three‐week values for the modified Glasgow prognostic score, C‐reactive protein‐albumin ratio, prognostic nutrition index, and advanced lung cancer inflammation index. The OS duration was significantly associated with the pre‐treatment values for the neutrophil‐to‐lymphocyte ratio and advanced lung cancer inflammation index, as well as the prognostic nutrition index at the end of induction therapy.

**Conclusions:**

Immunological and nutritional markers could be useful for predicting the outcomes of CIT for Japanese patients with advanced non‐small cell lung cancer. The timing of their evaluation may also be important.

**Key points:**

**Significant findings of the study:**

Overall survival in patients receiving first‐line chemoimmunotherapy for advanced lung cancer were associated with pretreatment values of neutrophil‐to‐lymphocyte ratio, advanced lung cancer inflammation index, and the prognostic nutrition index at the end of induction therapy.

**What this study adds:**

Repetitive evaluation of immunological and nutritional markers may be useful for guiding prognostication and treatment selection for Japanese patients with advanced lung cancer.

## Graphical abstract

Immunological and nutritional markers could be useful for predicting the outcomes of first‐line chemoimmunotherapy for Japanese patients with advanced non‐small cell lung cancer. Overall survival was significantly associated with the pretreatment values for the neutrophil‐to‐lymphocyte ratio and advanced lung cancer inflammation index, as well as the prognostic nutrition index at the end of induction therapy.

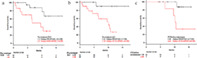

## Introduction

The introduction of immune checkpoint inhibitors (ICIs) has revolutionized the treatment strategy for non‐small cell lung cancer (NSCLC). Relative to docetaxel, and especially in NSCLC patients without oncogenic driver mutations or translocations, superior overall survival (OS) and progression‐free survival (PFS) are associated with second‐line ICI treatment using nivolumab^1^, ^2^ and pembrolizumab,^3^ which target programmed cell death‐1 (PD‐1), as well as atezolizumab,^4^ which targets programmed death ligand 1 (PD‐L1). However, rates of progressive disease (PD) are high after ICI treatment, with reported PD rates of 41% in nivolumab‐treated squamous (Sq) NSLCL,^1^ 44% in nivolumab‐treated non‐Sq NSCLC,^2^ and 42% in atezolizumab‐treated unselected NSCLC,^4^ regardless of tumor PD‐L1 expression, and these rates are consistently worse than those for docetaxel. Pembrolizumab monotherapy has been established as a standard first‐line treatment for NSCLC with PD‐L1 expression of ≥50% and no oncogenic driver mutations, based on astonishing increases in OS and PFS, relative to platinum doublet chemotherapy.^5^ However, the real‐world PD rate for pembrolizumab monotherapy has been reported to be as high as 28.7% in that patient population.^6^ Chemoimmunotherapy (CIT) combining pembrolizumab or atezolizumab with standard chemotherapy also provides superior OS, PFS, and PD rates (vs. chemotherapy alone) in patients with Sq NSCLC^7^ or non‐Sq NSCLC,^8^, ^9^, ^10^ regardless of their histological features. For example, reported PD rates for CIT include 6.1% for pembrolizumab plus carboplatin and nab‐paclitaxel,^7^ 8.8% for pembrolizumab plus cisplatin/carboplatin and pemetrexed,^8^ 3.9% for atezolizumab and bevacizumab plus carboplatin and paclitaxel,^9^ and 10.7% for atezolizumab plus carboplatin and nab‐paclitaxel.^10^

Upregulation of PD‐L1 expression is considered a predictive marker for nivolumab monotherapy in cases of pretreated non‐Sq NSCLC,^11^ as well as in NSCLC cases treated using first‐line pembrolizumab^5^ and second‐line pembrolizumab.^3^ However, it is unclear whether PD‐L1 expression is a predictive marker for CIT, as CIT provides superior PFS, regardless of PD‐L1 expression.^12^ Thus, it would be useful to identify predictive markers for CIT, which could help identify patients who are expected to benefit from CIT continuation and patients who should be switched to second‐line treatment. Previous studies have proposed predictive markers for ICI monotherapy based on the local and systemic host‐tumor interactions, which are closely related to the patient's immunological and nutritional statuses. Candidate predictive markers include the modified Glasgow prognostic score (mGPS),^13^, ^14^, ^15^, ^16^, ^17^, ^18^ C‐reactive protein (CRP)‐albumin ratio (CAR),^19^, ^20^, ^21^, ^22^ neutrophil‐to‐lymphocyte ratio (NLR),^23^, ^24^, ^25^, ^26^, ^27^, ^28^ prognostic nutrition index (PNI),^29^, ^30^ and advanced lung cancer inflammation index (ALI).^31^, ^32^ Interestingly, the host‐tumor interaction changes during anticancer treatment and during disease progression leading to cancer cachexia.^33^ Therefore, we aimed to retrospectively identify predictors of outcomes from first‐line CIT treatment for advanced NSCLC, and examine candidate markers at several time points during the treatment process.

## Methods

### Patients

This single‐center retrospective study evaluated 34 consecutive Japanese patients with NSCLC who received first‐line CIT between February 2019 and July 2020. NSCLC patients harboring driver mutations were excluded since they were treated with relevant tyrosine kinase inhibitors. The study protocol complied with the Declaration of Helsinki and was approved by the Ethics Committee of the Japanese Red Cross Kyoto Daini Hospital (January 24, 2020; S2019‐55). The requirement for informed consent was waived based on the retrospective analysis of anonymized patient data. Patients are allowed to opt‐out of research use of their data and related information is provided publicly on the hospital's website.

### CIT regimens

The treatment strategy was selected by the patient's physician according to the histological findings, the patient's Eastern Cooperative Oncology Group performance status (ECOG‐PS), comorbidities, and other factors. In theory, Sq NSCLC was treated using pembrolizumab plus carboplatin and nab‐paclitaxel (the KN‐407 regimen), while non‐Sq NSCLC was treated using pembrolizumab plus cisplatin/carboplatin and pemetrexed (the KN‐189 regimen), atezolizumab and bevacizumab plus carboplatin and paclitaxel (the IM150 regimen), or atezolizumab plus carboplatin and nab‐paclitaxel (the IM130 regimen). Patients diagnosed with NSCLC‐not otherwise specified (NSCLC‐NOS) were treated using pembrolizumab/atezolizumab plus carboplatin and nab‐paclitaxel.

Renal insufficiency was considered a major reason to avoid pemetrexed. Bevacizumab treatment was not administered to patients with a history of hemoptysis, tumor invasion into major vessels, cavitation in the primary tumor, or proteinuria (≥100 mg/dL). A maximum of four induction therapy cycles were followed by continuation maintenance therapy (CMT) using pembrolizumab monotherapy, pembrolizumab plus pemetrexed, or atezolizumab plus bevacizumab, based on the previously selected regimen.

### Assessments

The response of a tumor to treatment was assessed using the Response Evaluation Criteria in Solid Tumors (version 1.1). Chest radiography and computed tomography (CT) were used to evaluate treatment response, which was determined by the treating physicians and radiologists. First‐line CIT was discontinued when PD was detected, and treatment using CIT was not allowed after that point. Adverse events (AEs), including immune‐related AEs (irAEs), were evaluated using the Common Terminology Criteria for Adverse Events (version 5.0).

### Study variables

Previous reports were used to select cutoff values for the following immunological and nutritional markers: mGPS, CAR, NLR, PNI, and ALI. The mGPS was calculated as a score of 0 (CRP concentration of ≤1 mg/dL), score of 1 (CRP concentration of >1 mg/dL), or score of 2 (CRP concentration of >1 mg/dL and hypoalbuminemia [<3.5 g/dL]). The CAR was calculated as the ratio of CRP (mg/dL) to albumin (g/dL) and patients were grouped according to CAR values of <0.424 or ≥ 0.424. The NLR was calculated as the ratio of absolute neutrophil count (/μL) to lymphocyte count (/μL) and patients were grouped according to NLR values of <5 or ≥ 5. The PNI was calculated as 10 × albumin (g/dL) + 0.005 × absolute lymphocyte count (/μL) and patients were grouped according to PNI values of ≤40 or > 40. The ALI was calculated as (body mass index [BMI] × albumin) / NLR and patients were grouped according to ALI values of <18 or ≥ 18.

These markers were evaluated at the start of CIT, after three weeks, and at the end of the introduction therapy. Each marker was evaluated for associations with OS and PFS at the different time points, although patients who experienced PD within three weeks or who did not transition to CMT were excluded from the subsequent time point analyses.

### Statistical analysis

Statistical analyses were performed using EZR,^34^ which is a graphical user interface for R software (The R Foundation for Statistical Computing, Vienna, Austria). Baseline characteristics were compared using Pearson's chi‐squared test. The median PFS and OS intervals, with the corresponding 95% confidence intervals (CIs), and the objective response rate (ORR) were calculated. Curves for PFS and OS were evaluated using the Kaplan‐Meier method and log‐rank test. Univariate Cox proportional hazards regression analyses were performed for each potential marker, although multivariate analyses were not performed because of the small sample size. *P*‐values of <0.05 were considered statistically significant.

## Results

### Baseline patient characteristics

The 34 patients included five women and 29 men with a median age of 72 years (range: 55–81 years) (Table 1). The histological types were adenocarcinoma (non‐Sq NSCLC) for 14 patients, Sq NSCLC for 11 patients, NSCLC‐NOS for eight patients, and pleomorphic carcinoma for one patient. The PD‐L1 status was available for 30 of the 34 patients. The treatment regimens were the KN‐407 regimen for 19 patients, the KN‐189 regimen for 10 patients, the IM150 regimen for one patient, and the IM130 for four patients.

**Table 1:** Baseline patient characteristics

Characteristics	n (%)
Age	
Median (range)	72 (55–81)
Sex	
Male	29 (85.3)
Female	5 (14.7)
Histology	
Adenocarcinoma	14 (41.2)
Squamous cell carcinoma	11 (32.4)
NSCLC‐NOS	8 (23.5)
Pleomorphic carcinoma	1 (2.9)
ECOG performance status	
0–1	33 (97.1)
2	1 (2.9)
PD‐L1 TPS	
<1%	3 (8.8)
1%–49%	10 (29.4)
≥50%	17 (50)
Unknown	4 (11.8)
Values used in the predictive markers	
Albumin (g/dL, range)	3.2 (1.8–4.1)
CRP (mg/dL, range)	1.5 (0.05–20.42)
BMI (range)	21.39 (15.71–28.45)
Neutrophil counts (/μL, range)	5287 (2239–17 622)
Lymphocyte counts (/μL, range)	1289 (198–2632)
Regimen	
KEYNOTE‐189	10 (29.4)
KEYNOTE‐407	19 (55.9)
IMpower150	1 (2.9)
IMpower130	4 (11.8)

BMI, body mass index; CRP, C‐reactive protein; ECOG, Eastern Cooperative Oncology Group; NOS, not otherwise specified; NSCLC, non‐small cell lung cancer; PD‐L1, programmed death ligand 1; TPS, tumor proportion score.

### ORR, PFS, and OS outcomes

**Figure 1:**
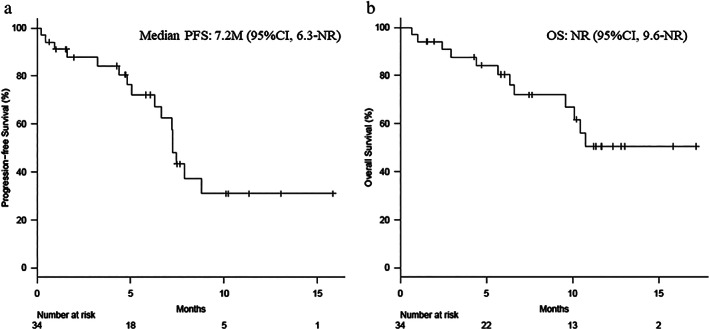
Kaplan‐Meier estimates of (**a**) progression‐free survival (PFS) and (**b**) overall survival (OS) among all patients. The median PFS was 7.2 months (95% confidence interval [CI]: 6.3 months–not reached) and the median OS was not reached (95% CI: 9.6 months–not reached).

Among the 34 patients, the treatment responses were classified as partial response in 14 patients (41.2%), stable disease in 10 patients (29.4%), PD in four patients (11.8%), and not evaluable in six patients (17.6%). The median PFS was 7.2 months (95% CI: 6.3 months–not reached) and the median OS was not reached (95% CI: 9.6 months–not reached). The Kaplan‐Meier curves for PFS and OS among all patients are shown in Fig 1. The median observation period was 7.1 months (range: 0.67–17.4 months).

### Cycles of CIT and the second line treatments

During the observation period, the median cycle of induction therapy was four (range: 1–4), and that of CMT was two (range: 0–15). After disease progression in 23 patients, the following treatments were added; docetaxel in four, carboplatin plus S‐1 in two, S‐1 in two, docetaxel plus ramucirumab, carboplatin plus pemetrexed, carboplatin plus nab‐paclitaxel, carboplatin plus paclitaxel, pembrolizumab in one each, with the remaining 10 patients selecting best supportive care.

### Constituent values used in the predictive markers

The constituent values (median and range) used in the predictive markers at baseline were as follows (Table 1): albumin, 3.2 g/dL (1.8–4.1); CRP, 1.5 (0.05–20.42); BMI, 21.39 (15.71–28.45); neutrophil counts, 5287/μL (2239–17 622); lymphocyte counts, 1289/μL (198–2632). The values after three weeks were as follows: albumin, 3.4 g/dL (2.2–4.2); CRP, 1.35 (0.07–11.91); BMI, 21.43 (14.59–27.38); neutrophil counts, 2799/μL (1097–8927); lymphocyte counts, 1324/μL (445–3332). The values before maintenance were as follows: albumin, 3.7 g/dL (2.8–4.4); CRP, 0.3 (0.06–4.37); BMI, 21.29 (14.96–27.14); neutrophil counts, 2075/μL (1114–5625); lymphocyte counts, 1171/μL (554–2303). The values of each constituent obtained at three time points did not show statistically significant correlation with PFS and OS. Since the variables of immunological and nutritional markers investigated in the current study had been proposed to provide a better prognosis of the outcomes by enhancing these constituents, these constituents were excluded from the current investigation.

### Relationships between the various markers, PFS, and OS

Univariate analyses were performed for baseline characteristics, the occurrence of irAEs, and the markers at the three time points (Table 2). Among 34 patients, three patients progressed within three weeks and 11 patients did not transition to CMT.

**Table 2:** Univariate analyses of predictors of progression‐free survival (PFS) and overall survival (OS)

	PFS		OS	
	HR (95% CI)	*P*‐value	HR (95% CI)	*P*‐value
Age of ≥70 years (*n* = 20)	2.21 (0.79–6.21)	0.133	2.00 (0.60–6.71)	0.260
Male sex (*n* = 29)	1.63 (0.37–7.20)	0.521	2.39 (0.31–18.6)	0.405
Smoker (*n* = 31)	1.09 (0.14–8.43)	0.933	75 640 000 (0–N/A)	0.998
ECOG‐PS of ≥2 (*n* = 1)	0 (0–N/A)	0.999	**32.5 (2.03–520)**	**0.014**
PD‐L1 of <50% (*n* = 13)	2.39 (0.83–6.93)	0.107	2.84 (0.82–9.78)	0.098
irAEs (*n* = 19)	0.77 (0.26–2.29)	0.639	0.33 (0.10–1.07)	0.065
Before treatment *(n = 34)*				
mGPS of ≥1 (*n* = 24)	3.34 (0.94–11.9)	0.061	6.58 (0.85–51.1)	0.071
CAR of ≥0.424 (*n* = 17)	2.19 (0.81–5.96)	0.125	1.34 (0.43–4.20)	0.611
NLR of ≥5 (*n* = 14)	**3.73 (1.29–10.8)**	**0.015**	**3.68 (1.11–12.3)**	**0.034**
PNI of ≤40 (*n* = 19)	1.59 (0.57–4.38)	0.375	1.80 (0.54–5.98)	0.340
ALI of <18 (*n* = 20)	2.16 (0.74–6.28)	0.157	**9.84 (1.26–76.5)**	**0.029**
After 3 weeks *(n = 31)*				
mGPS of ≥1 (*n* = 21)	**4.17 (1.15–15.1)**	**0.030**	3.08 (0.63–15.0)	0.163
CAR of ≥0.424 (*n* = 15)	**4.86 (1.50–15.7)**	**0.008**	4.36 (0.90–21.0)	0.067
NLR of ≥5 (*n* = 5)	2.72 (0.83–8.92)	0.100	2.40 (0.59–9.70)	0.219
PNI of ≤40 (*n* = 14)	**3.03 (1.06–8.66)**	**0.039**	2.20 (0.59–8.19)	0.242
ALI of <18 (*n* = 10)	**3.69 (1.29–10.6)**	**0.015**	2.00 (0.53–7.46)	0.304
Before maintenance *(n = 23)*				
mGPS of ≥1 (*n* = 8)	2.62 (0.78–8.79)	0.118	1.95 (0.32–11.7)	0.466
CAR of ≥0.424 (*n* = 4)	1.00 (0.21–4.65)	0.997	0 (0–N/A)	0.999
NLR of ≥5 (*n* = 1)	1.86 (0.23–15.2)	0.564	0 (0–N/A)	0.999
PNI of ≤40 (*n* = 7)	1.79 (0.54–5.88)	0.341	**9.84 (1.08–89.5)**	**0.042**
ALI of <18 (*n* = 2)	2.45 (0.49–12.3)	0.275	0 (0–N/A)	0.999

Bold values indicate statistical significance. ALI, advanced lung cancer inflammation index; CAR, C‐reactive protein–albumin ratio; ECOG‐PS, Eastern Cooperative Oncology Group performance status; HR, hazard ratio; irAE, immune‐related adverse event; mGPS, modified Glasgow prognostic score; NLR, neutrophil‐to‐lymphocyte ratio; PNI, prognostic nutrition index; PD‐L1, programmed death ligand 1.

**Figure 2:**
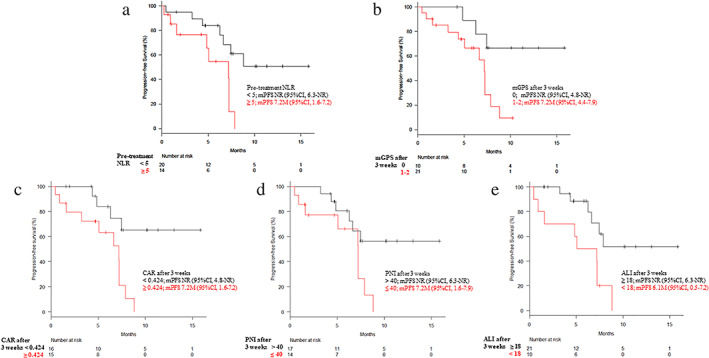
Kaplan‐Meier estimates of progression‐free survival (PFS) according to (**a**) pretreatment neutrophil‐to‐lymphocyte ratio (NLR) and three‐week values for (**b**) modified Glasgow prognostic score (mGPS), (**c**) C‐reactive protein‐albumin ratio (CAR), (**d**) prognostic nutrition index (PNI), and (**e**) advanced lung cancer inflammation index (ALI). Longer median PFS values were associated with an NLR of <5, mGPS of 0, CAR of <0.424, PNI of >40, and ALI of ≥18. CI, confidence interval; NR, not reached.

The PFS outcomes were significantly associated with the baseline NLR and the three‐week values for mGPS, CAR, PNI, and ALI (Fig 2). The median PFS in patients with baseline NLR values of ≥5 and <5 was 7.2 months (95% CI: 1.6–7.2 months) and not reached (95% CI: 6.3 months–not reached), respectively (hazard ratio [HR]: 3.73). The median PFS in patients with mGPS scores of 1–2 and 0 after three weeks was 7.2 months (95% CI: 4.4–7.9 months) and not reached (95% CI: 4.8 months–not reached), respectively (HR: 4.17). The median PFS in patients with CAR values of ≥0.424 and < 0.424 after three weeks was 7.2 months (95% CI: 1.6–7.2 months) and not reached (95% CI: 4.8 months–not reached), respectively (HR: 4.86). The median PFS in patients with PNI values of ≤40 and > 40 after three weeks was 7.2 months (95% CI: 1.6–7.9 months) and not reached (95% CI: 6.3 months–not reached), respectively (HR: 3.03). The median PFS in patients with ALI value of <18 and ≥ 18 after three weeks was 6.1 months (95% CI: 0.5–7.2 months) and not reached (95% CI: 6.3 months–not reached), respectively (HR: 3.69). Figure 2 shows clear differences in the Kaplan‐Meier curves for PFS according to the different predictive markers from the univariate analyses.

**Figure 3:**
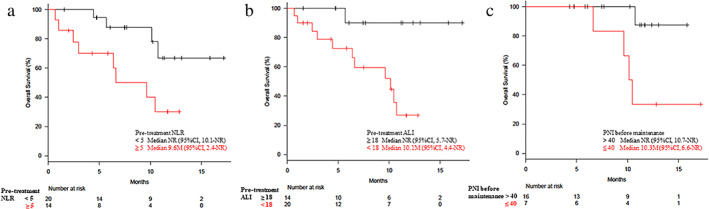
Kaplan‐Meier estimates of overall survival (OS) according to (**a**) pretreatment neutrophil‐to‐lymphocyte ratio (NLR), (**b**) pretreatment advanced lung cancer inflammation index (ALI), and (**c**) prognostic nutrition index (PNI) at the end of induction therapy. Longer median OS values were associated with a NLR of <5, an ALI of ≥18, and a PNI of >40. CI, confidence interval; NR, not reached.

The OS outcomes were significantly associated with the baseline ECOG‐PS, the pretreatment values for NLR and ALI, and the PNI at the end of induction therapy (before CMT). The median OS in patients with baseline NLR values of ≥5 and < 5 was 9.6 months (95% CI: 2.4 months–not reached) and not reached (95% CI: 10.1 months–not reached), respectively (hazard ratio [HR]: 3.68). The median OS in patients with baseline ALI value of <18 and ≥ 18 was 10.1 months (95% CI: 4.4 months–not reached) and not reached (95% CI: 5.7 months–not reached), respectively (HR: 9.84). The median OS in patients with PNI values of ≤40 and > 40 at the end of induction therapy (before maintenance therapy) was 10.3 months (95% CI: 6.6 months–not reached) and not reached (95% CI: 10.7 months–not reached), respectively (HR: 9.84). Figure 3 shows clear differences in the Kaplan‐Meier curves for OS according to the different predictive markers from the univariate analyses.

### Adverse events

Any‐grade AEs were observed for 25 patients (73.5%) and grade 3–4 AEs were observed for 18 patients (52.9%). It was difficult to distinguish between chemotherapy‐related AEs and irAEs in some cases (Table 3).

**Table 3:** Adverse events

	Any grade (%)	Grades 3–5 (%)
Any event	25 (73.5)	18 (52.9)
Neutropenia	10 (29.4)	6 (17.6)
Skin rash	8 (23.5)	1 (2.9)
Pneumonitis	6 (17.6)	4 (11.8)
Nausea	5 (14.7)	1 (2.9)
Liver dysfunction	5 (14.7)	0 (0)
Renal dysfunction, nephritis	3 (8.8)	1 (2.9)
Peripheral neuropathy	3 (8.8)	1 (2.9)
Hair loss	3 (8.8)	0 (0)
Stevens‐Johnson syndrome	2 (5.9)	2 (5.9)
Vogt‐Koyanagi‐Harada disease	1 (2.9)	1 (2.9)
Colitis	1 (2.9)	1 (2.9)
Diabetes	1 (2.9)	1 (2.9)
Adrenal insufficiency	1 (2.9)	1 (2.9)
Heart failure	1 (2.9)	1 (2.9)
Febrile neutropenia	1 (2.9)	1 (2.9)
Anemia	1 (2.9)	1 (2.9)
Thrombocytopenia	1 (2.9)	0 (0)
Fever	1 (2.9)	0 (0)
Hypothyroidism	1 (2.9)	0 (0)
Taste disorder	1 (2.9)	0 (0)
Fundus bleeding	1 (2.9)	0 (0)

## Discussion

The introduction of ICIs has improved treatment outcomes among patients with NSCLC, and the introduction of first‐line CIT has also provided additional benefits. The predictive values of immunological and nutritional markers have been rigorously investigated in ICI monotherapy in pretreated NSCLC.^28^, ^31^, ^32^ However, the usefulness of these markers during CIT has not previously been elucidated. Considering the different as well as homologous mechanism between ICI monotherapy and CIT, a multifaceted analysis of these markers during the first‐line CIT was needed.

The cancer‐immune cycle^35^ is widely recognized as the conceptual mechanism underlying the efficacy of immunotherapy, which has been further extended to three theoretical profiles regarding the immune response: immune‐inflamed tumor, immune‐excluded tumor, and immune‐desert tumor.^36^ In this context, NSCLC with an inflamed profile is considered the optimal target for anti‐PD‐1/PD‐L1 treatment, and PD‐L1 expression on tumor cells is considered an important marker, despite it being difficult to precisely assign patients to these three theoretical profiles. Pembrolizumab monotherapy provides significantly superior ORR, PFS, and OS for patients with NSCLC and PD‐L1 expression of ≥50%,^5^ although high rates of PD have been reported (>20% regardless of race), with rates reaching 28.7% among French patients^6^ and 24.2% among Japanese patients.^37^ Thus, high tumor PD‐L1 expression is not always associated with a response to pembrolizumab monotherapy and it is possible that patients who do not benefit from ICI could have immune‐desert or immune‐excluded tumors. Nevertheless, anti‐PD‐1/PD‐L1 treatments might still be effective for immune‐desert or immune‐excluded tumors by activating an impaired priming phase^38^ and improving the trafficking of cytotoxic T cells to the tumor via the antivascular endothelial growth factor (VEGF) effects of bevacizumab.^39^ Cytotoxic chemotherapy is considered a pivotal factor in activating the priming phase through tumor‐derived neoantigen release,^38^ as well as by sensitizing cancer cells to activated T cells via pemetrexed‐based regimens.^40^ Anti‐VEGF therapy may also contribute to improved treatment efficacy through immune‐modulating effects, including dendritic cell activation and enhancing the T cell response.^41^

Previous studies regarding predictive markers for CIT have focused on pretreatment tumor PD‐L1 expression.^12^, ^42^ Although PD‐L1 expression is important for predicting outcomes, the PD‐L1 expression status may be unclear in cases where only a small specimen was obtained, and is not suitable for repetitive evaluation. Thus, we considered readily available predictive markers that reflect host‐tumor interactions. Furthermore, the immunological and nutritional status of a patient can vary during anticancer treatment, which prompted us to consider these markers at three time points: at the start of CIT, after three weeks, and at the end of induction therapy. We anticipated that the values from the end of induction therapy would predict the outcomes because they reflect the host‐tumor interaction before the maintenance therapy, whereas ICI plays a dominant role in achieving a durable response. However, PFS was associated with the pretreatment NLR value and the three‐week values for mGPS, CAR, PNI, and ALI, while OS was associated with the pretreatment values for NLR and ALI, as well as the PNI value at the end of induction therapy. It is possible that the lack of a significant association between post‐treatment NLR and survival was related to the NLR not considering measures of nutritional status, such as albumin and BMI. In contrast, the mGPS, CAR, PNI, and ALI values reflect the patient's nutritional status and can change during treatment, which may make these markers superior for predicting PFS. It is difficult to explain why OS was associated with the pretreatment NLR and ALI values and the PNI value at the end of induction therapy. However, the NLR and ALI consider lymphocyte and neutrophil counts, which provide a good reflection of the patient's immunological status (vs. other markers), as lymphocytes lead to immune tumor control via suppressive effects,^43^, ^44^ while neutrophils induce the production of pro‐inflammatory cytokines and chemokines that promote tumor proliferation, invasion, and angiogenesis.^45^, ^46^ The PNI considers lymphocyte count and albumin concentration (reflecting nutritional status), and these factors also fluctuate during induction therapy, which may suggest that the patient's immunological and nutritional statuses at the end of induction therapy can predict OS.

This study has several limitations. First, the retrospective single‐center design is prone to bias. Second, the small sample size is another potential source of bias, including relatively high rate of NSCLC‐NOS observed in the current study. Third, we selected cutoff values for the various predictive markers from previous reports, rather than those based on our own analyses. Therefore, the markers' utilities and optimal cutoff values should be clarified in larger prospective studies.

In conclusion, the present study revealed that PFS and OS might be related to the immunological and nutritional statuses of patients before and during CIT for advanced NSCLC. For example, the PFS outcomes were significantly associated with the baseline NLR and the three‐week values for mGPS, CAR, PNI, and ALI. The OS outcomes were significantly associated with the baseline ECOG‐PS, the pretreatment values for NLR and ALI, and the PNI at the end of induction therapy (before CMT). This information may help guide prognostication and treatment selection for Japanese patients with advanced lung cancer, and help identify patients who should proceed to second‐line treatment.

## Disclosure

The authors declare that they have no conflicts of interest.
